# Model Membrane Platforms for Biomedicine: Case Study on Antiviral Drug Development

**DOI:** 10.1007/s13758-011-0018-2

**Published:** 2012-02-11

**Authors:** Joshua A. Jackman, Nam-Joon Cho

**Affiliations:** 1 School of Materials Science and Engineering, Nanyang Technological University, 50 Nanyang Avenue, Singapore, 639798 Singapore; 2 School of Biological Sciences, Nanyang Technological University, 60 Nanyang Drive, Singapore, 637551 Singapore; 3 Centre for Biomimetic Sensor Science, Nanyang Technological University, 50 Nanyang Drive, Singapore, 637553 Singapore; 4 Division of Gastroenterology and Hepatology, Department of Medicine, Stanford University, 269 Campus Drive, Stanford, CA 94305 USA

**Keywords:** Lipid Vesicle, Quartz Crystal Microbalance With Dissipation, Arbidol, Planar Bilayer, Lipid Envelope

## Abstract

As one of the most important interfaces in cellular systems, biological membranes have essential functions in many activities such as cellular protection and signaling. Beyond their direct functions, they also serve as scaffolds to support the association of proteins involved in structural support, adhesion, and transport. Unfortunately, biological processes sometimes malfunction and require therapeutic intervention. For those processes which occur within or upon membranes, it is oftentimes difficult to study the mechanism in a biologically relevant, membranous environment. Therefore, the identification of direct therapeutic targets is challenging. In order to overcome this barrier, engineering strategies offer a new approach to interrogate biological activities at membrane interfaces by analyzing them through the principles of the interfacial sciences. Since membranes are complex biological interfaces, the development of simplified model systems which mimic important properties of membranes can enable fundamental characterization of interaction parameters for such processes. We have selected the hepatitis C virus (HCV) as a model viral pathogen to demonstrate how model membrane platforms can aid antiviral drug discovery and development. Responsible for generating the genomic diversity that makes treating HCV infection so difficult, viral replication represents an ideal step in the virus life cycle for therapeutic intervention. To target HCV genome replication, the interaction of viral proteins with model membrane platforms has served as a useful strategy for target identification and characterization. In this review article, we demonstrate how engineering approaches have led to the discovery of a new functional activity encoded within the HCV nonstructural 5A protein. Specifically, its N-terminal amphipathic, α-helix (AH) can rupture lipid vesicles in a size-dependent manner. While this activity has a number of exciting biotechnology and biomedical applications, arguably the most promising one is in antiviral medicine. Based on the similarities between lipid vesicles and the lipid envelopes of virus particles, experimental findings from model membrane platforms led to the prediction that a range of medically important viruses might be susceptible to rupturing treatment with synthetic AH peptide. This hypothesis was tested and validated by molecular virology studies. Broad-spectrum antiviral activity of the AH peptide has been identified against HCV, HIV, herpes simplex virus, and dengue virus, and many more deadly pathogens. As a result, the AH peptide is the first in class of broad-spectrum, lipid envelope-rupturing antiviral agents, and has entered the drug pipeline. In summary, engineering strategies break down complex biological systems into simplified biomimetic models that recapitulate the most important parameters. This approach is particularly advantageous for membrane-associated biological processes because model membrane platforms provide more direct characterization of target interactions than is possible with other methods. Consequently, model membrane platforms hold great promise for solving important biomedical problems and speeding up the translation of biological knowledge into clinical applications.

## Introduction

Biological membranes represent one of the most important interfaces in cellular systems [1–3]. From serving as a physical barrier [4] to regulating signal transduction pathways [2], they play a key role in cellular protection and homeostasis. Beyond their own direct functions, membranes are also scaffolds upon which proteins associate to stabilize cellular structure [5], promote adhesion [6], and direct transport processes [7], among other functions. As one measure of the membrane interface’s biomedical importance, around 60% of approved drug targets are membrane-associated proteins [8]. In addition, many viral pathogens take advantage of membranes as part of their life cycle. Specifically, membranes serve as a platform for viral proteins to bind to the host cell [9] as well as to organize themselves into complexes that catalyze critical synthesis activities during viral genome replication [10].

Given all of these events that take place at the membrane interface, there are many possibilities for therapeutic intervention to target disease-related processes such as abnormal signaling [11], viral membrane fusion [12], and inflammatory cascades [13]. Beyond the wide range of membrane-associated processes amenable to molecular targeting, there can also be multiple strategies against one target. For example, ion channels embedded in lipid membranes may either be stimulated or inhibited by electrical force, mechanical force, or chemical cues [14]. Other therapeutic possibilities include targeting the lipid membrane to modulate its physical properties including permeability [15], as well as disrupting the enzymatic activities of membrane-associated proteins [16] or their interactions with other macromolecule components [17]. Regardless of the biomedical problem, it is necessary to understand the fundamental molecular mechanisms of the target interaction.

Continuing progress in the development of biological tools has led to major advances for determining the structure and function of biological macromolecules. Biophysical methods such as nuclear magnetic resonance (NMR) and electron paramagnetic resonance (EPR) spectroscopy have enabled structural characterization of proteins in their membrane-associated states [18, 19]. This information has significantly improved fundamental understanding of the structure–function relationships of membrane-associated proteins, as well as aid in the identification of new therapeutic targets. However, to date, a major challenge has been monitoring the dynamics of membrane-protein interactions as well as protein–protein interactions and enzymatic processes at membrane interfaces.

To overcome this challenge, a common tactic has been to design soluble forms of analyte proteins that can be studied directly in solution [20], or in the presence of membrane-mimicking components such as detergents [21]. Although the enzymatic activities of these recombinant proteins can typically be monitored [22, 23], no information is gained about the interaction of the protein with its host membrane environment, how this interaction affects its function, or how the step of membrane association may be targeted. As such, the amount of information that can be gained about the target interaction is limited when using non-native systems.

Engineering strategies offer a promising approach to study the interfacial science principles of membrane-associated biological activities in physiologically relevant settings. A central theme in the engineering sciences is the fundamental characterization of a model biological system by defining the necessary parameters and then systematically evaluating them. Consequently, this approach can help to understand the self-assembly and functional activity of biological systems by easily changing parameters. To facilitate characterization work, model systems are often studied with surface-sensitive analytical tools [24]. A range of techniques exist to probe biological thin film interfaces, and can measure acoustic [25], optical [26], spectroscopic [27], electrical [28], chemical [29], and morphological properties [30]. With this wealth of options, there are many analytical strategies to choose from in order to characterize the specific target interaction.

Regardless of the characterization methods, it is important to design an appropriate model system that mimics important structural properties of the target interaction [24, 31–33]. Importantly, for the study of membrane-associated biological processes, model membranes enjoy the advantage of self-assembly formation [31, 34], which permits facile adjustment of system parameters. As such, bottom-up design has enabled the development of model membrane systems for probing a wide range of biological processes such as cell adhesion, membrane fusion, and cellular signaling, and there are many excellent reviews already in the field [35–39]. However, there has been limited demonstration thus far of how knowledge gained from these approaches can be translated into clinically impactful biomedical applications. In this review article, we establish a framework to demonstrate how engineering strategies can leverage the sensing advantages of model membrane systems to yield novel therapies that are poised to enter the drug pipeline and solve challenging biomedical problems.

One of the most serious global health problems is the urgent need to combat infectious diseases. With the emergence of new infectious diseases as well as the reemergence of others, there are a number of socioeconomic, evolutionary, and environmental, and molecular biology issues which hinder the development of more effective diagnostic and therapeutic options. Indeed, one of the biggest hurdles is the need to identify new molecular targets in order to counter the emergence of drug-resistant pathogens. As a case study example of a viral pathogen target, we have selected the hepatitis C virus (HCV) and discuss how engineering strategies identified a previously unknown functional activity encoded within the HCV nonstructural 5A (NS5A) protein. By employing a model membrane platform to mimic the host cell membrane interface upon which viral genome replication occurs, a striking ability to lyse lipid vesicles was discovered within the NS5A N-terminal amphipathic, α-helix (AH) [40–43].

Because lipid vesicles also serve as a simplified model for the lipid envelope of virus particles, mechanistic studies predicted that a range of medically important viruses might be susceptible to rupturing treatment with a synthetic AH peptide [41]. Importantly, these predictions were tested and validated by molecular virology experiments which demonstrated AH peptide has broad-spectrum antiviral activity against HCV, HIV, herpes simplex virus, and dengue virus, as well as many more deadly pathogens [44–46]. As such, the AH peptide represents the first in class of lipid envelope-rupturing antiviral agents. Beyond the identification of this one drug candidate, these studies have also led to the development of a new testing platform to rapidly characterize antiviral drug candidates in minutes. Taken together, model membrane technology demonstrates significant potential for biomedicine, and is yielding new biotechnology and clinical applications to solve a range of important biomedical problems.

## Challenges of Hepatitis C Drug Development

**Fig. 1:**
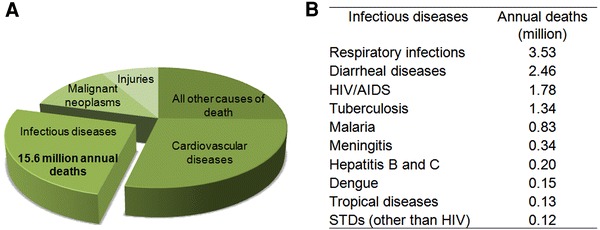
Global health challenges of infectious diseases. **a** Over 15 million (>25%) of the 57 million annual deaths worldwide are the direct result of infectious diseases. **b** Several classes of infectious diseases cause the deaths of hundreds of thousands of people each year. Barriers to the successful treatment of these diseases include lack of basic resources, insufficient preventive measures, and ineffective therapies. Figure is adapted and updated from Ref. [47]. Original data was published in *The World Health Report 2008* (World Health Organization, Genève, 2008)

Throughout history, the impact of infectious diseases on human society has been staggering, reaching across numerous sectors including economy, sociology, healthcare, politics, and education [47]. At present, over 25% of the approximately 57 million annual deaths in the world are the direct result of infectious diseases [47] (Fig. 1a). Within this number, several classes of disease individually cause the deaths of hundreds of thousands or even millions of people per year [47] (Fig. 1b). Several million more deaths occur each year from complications of chronic infections [47]. Despite advances in treatment options, significant work remains towards the eradication of infectious diseases.

Thus far, the principal antiviral therapeutic strategy has been vaccination [48]. While this strategy has proven effective for eradicating viral pathogens such as polio and smallpox, vaccine development for several important viral diseases including respiratory tract viruses, human papilloma viruses, herpes viruses, and haemorrhagic fever viruses has been unsuccessful [48]. Moreover, while vaccines are available for influenza virus types A and B and for Hepatitis B virus, effective treatment requires additional antiviral drugs, which are still prone to the emergence of viral resistance as well as variable tolerance and efficacies [48]. Most notably, there is no promising vaccine in the pipeline for either HCV or HIV. Nonetheless, these viral pathogens are two of the deadliest. Over 40 million people worldwide are infected with HIV, while an astounding 170 million people are infected with HCV [49]. Given the large-scale impact of these viral pathogens, we have selected HCV as a prototype case to demonstrate how model membranes offer a platform to target membrane-associated steps in the virus life cycle in order to catalyze the development of more powerful antiviral drugs.

**Fig. 2:**
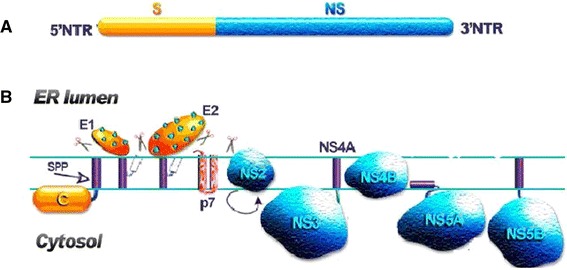
Organization of HCV genome and implications for polyprotein processing. **a** The HCV genome is a single-stranded RNA molecule that encodes a polyprotein of ~3,010 amino acids. The RNA structures of 5′ and 3′ untranslated regions (UTR) are highly conserved and essential for polyprotein translation and genome replication. S and NS correspond to genomic regions that code for structural and nonstructural proteins, respectively. **b** Location of HCV proteins relative to the ER membrane. HCV polyprotein is cotranslationally and posttranslationally processed by cellular and viral proteases to produce 10 mature proteins. Figure is adapted and modified from Ref. [50]

HCV is a member of the Flaviviridae family, which contains the Hepacivirus, Flavivirus and Pestivirus genera [50]. While there is a diversity of viruses within this family—dengue, classical swine fever, and viral encephalitis are just a few examples—there are also important common features: they are all small, lipid-enveloped RNA viruses and have many analogous steps in the viral life cycle including host cell entry, viral polyprotein processing, and viral genome replication [51]. Indeed, similar to other Flaviviridae members, HCV is a positive single-stranded RNA virus with a 9.6 kb genome that encodes for a 3010-amino acid long polyprotein [52] (Fig. 2a). Upon translation by the host cell machinery, the nascent polyprotein is then processed by cellular and viral proteases to produce two classes of mature protein: (1) structural components of the mature viron; and (2) nonstructural (NS) proteins that play important roles in viral genome replication [53] (Fig. 2b). Because replication of the HCV genome is error-prone, this process creates genomic diversity that hinders effective treatment of HCV infection. Targeting the NS proteins that mediate replication is an attractive pharmaceutical strategy to halt HCV infection. As there are a wide range of NS proteins, a summary of their properties including membrane association, function, and status as a drug target is presented in Table 1.

**Table 1:** Functional targeting of the HCV replicase

Protein	Membrane associating region	Replicase function(s)	Functional targets	Necessary for replication	Drug pipeline
NS2	At least one transmembrane domain	(1) NS2–NS3 proteolytic cleavage	(1) Transmembrane domains	No^a^	Preclinical
			(2) Protease active site		
			(3) Dimerization motif		
NS3	None	(1) Serine protease domain releases NS proteins downstream	(1) Protease active site	Yes	Phase lll/IV^b^
			(2) Helicase active site		
		(2) Helicase domain unwinds dsRNA/ssRNA			
			(3) NTPase active site		
			(4) RNA-binding region		
			(5) NS4A- and NS5B-interaction regions		
NS4A	N-terminal transmembrane domain	(1) NS3 cofactor for proper folding and membrane localization	(1) N-terminal transmembrane domain	Yes	ibid
NS4B	N-terminal AH	(1) Induces membranous web formation	(1) N-terminal AH	Yes	Phase I
			(2) Internal AH		
			(3) RNA-binding region		
			(4) Oligomerization motif		
NS5A	N-terminal AH	Unknown^c^	(1) N-terminal AH	Yes	Phase I^d^
			(2) Domain I dimerization motif for RNA binding		
			(3) Core protein interaction region		
NS5B	C-terminal anchor region	(1) Synthesis of viral genome RNA	(1) Polymerase active site	Yes	Phase II
			(2) Allosteric binding sites		
			(3) NS3- and NS5A-interaction regions		
			(4) RNA-binding region		
			(5) Oligomerization motif		

Like other positive-strand RNA viruses, HCV genome replication occurs in intimate association with host cell or host cell-derived intracellular membranes. HCV NS proteins play critical roles in the replication process, and are responsible for replicase assembly and function, including synthesis of the viral genome. Targeting critical functions of NS proteins is an attractive pharmaceutical strategy to halt HCV infection. A summary of NS proteins including their membrane-associating domain, function, necessity for viral replication and status as a drug target is presented. Information presented in the table was collected from Refs. [50, 51, 53, 58, 119]

^a^Proteolytic cleavage of the NS2–NS3 polyprotein is necessary prior to replication

^b^Clinical stage NS3/NS4A drug candidates exclusively target protease activity

^c^NSSA phosphorylation is proposed to regulate the switch between HCV genome replication and virion assembly

^d^BMS-790052 has a picomolar EC_50_. High therapeutic index and synergetic cocktail effects in Vitro

Like other positive-strand RNA viruses, HCV genome replication occurs in close association with host cell-derived membranes, particularly from the endoplasmic reticulum (ER). While genome replication of some viruses occurs on the surface of preexisting vesicular membranes [54], other viruses, including HCV, promote the formation of de novo membrane structures [55, 56]. Most of the HCV NS proteins interact together on a membranous platform (termed the “membranous web”) to form the replicase, a multi-protein complex that directs RNA polymerase activity [55, 57]. As inhibitors of the NS3 protease and NS5B polymerase are entering late-stage clinical trials [58] (see Table 1), it is already becoming clear that additional targets in the HCV replicase need to be identified to minimize the effects of viral resistance on successful clinical treatment [59]. Therefore, a major area of research in the molecular virology of HCV is the identification of new targets for pharmacologic inhibition.

In recent years, significant focus has been directed at the HCV NS5A protein. While this protein is implicated in regulating host cell protein activities and modulating virus sensitivity to interferon treatment, it has no known enzymatic function [60–62]. Nevertheless, NS5A is a key component of the HCV replicase complex and is necessary for viral replication [63]. Given that HCV replicase activity occurs at the membrane interface, it is interesting that NS5A possesses an N-terminal amphipathic, α-helix (AH) which is necessary for proper cytoplasmic membrane localization of NS5A in the host cell [64]. Indeed, genetic mutation analysis confirmed that the NS5A AH is necessary for HCV replication [63]. Together, the necessity of NS5A AH for membrane association and viral genome replication suggests that targeting membrane association of NS5A would represent a prime therapeutic target to inhibit replication [65]. Compared to other HCV targets that are prone to viral resistance, the NS5A AH possesses significant sequence homology across all HCV isolates identified to date [64]. Thus, its amphipathic, membrane-associating property is a conserved functional target. The design of a model system to investigate membrane association of the NS5A AH might lead to a viable inhibition strategy.

## Engineering Strategy to Mimic Biological Membranes

**Fig. 3:**
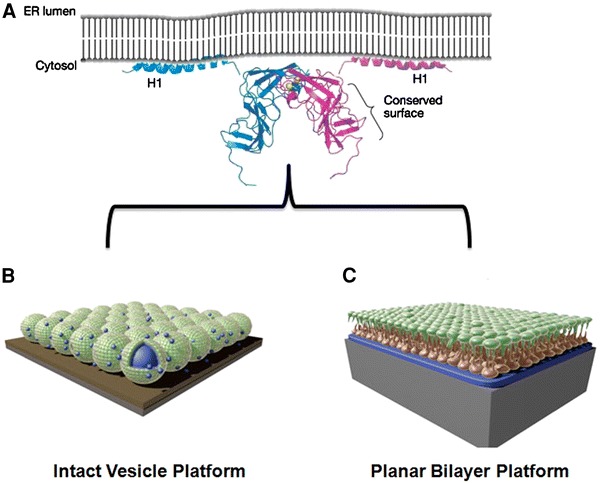
Model membrane platforms to mimic HCV replicase setting. To design a model system for functional characterization studies, it is necessary to identify the key components of the biological system. The HCV replicase assembles on endoplasmic reticulum (ER) membranes. In this context, the lipid bilayer of the ER membrane supports assembly and function of the replicase complex by hosting amphipathic helices found in NS proteins. Therefore, model membrane platforms based on a lipid bilayer are useful for functional analysis and pharmaceutical targeting of the HCV replicase. **a** Model orientation of the HCV NS5A protein relative to the ER membrane. Although membrane association the NS5A protein is necessary for viral replication, it possesses no known enzymatic function. Therefore, targeting the N-terminal AH—which is necessary for membrane association—represents an attractive inhibition strategy. **b** Intact vesicle platform is a monolayer of adsorbed, unruptured vesicles. The vesicle size distribution can control the morphological and viscoelastic properties of the film. **c** Planar bilayer platform is a rigid film formed by the adsorption and rupture of lipid vesicles on certain hydrophilic substrates. Figure is adapted and modified from Refs. [66, 82]

As shown in Fig. 3a, a representative model is presented for membrane association of NS5A domain I, which contains the N-terminal AH. While domain I is proposed to associate to lipid membranes as a dimer [66], NMR studies in detergent systems have shown that the AH region interacts with lipid membranes in a monotypic fashion [67]. The AH partially inserts itself into the membrane in a parallel orientation [67]. To follow up on this structural characterization work, there has been an increasing focus on developing methods to study the binding kinetics of NS5A AH membrane association. In this regard, model membrane platforms offer several unique advantages.

Model membranes are simplified versions of cellular membranes that are based on lipid bilayer assemblies [34]. For biological studies, the design emphasis is on mimicking the most fundamental properties of membranes including bilayer environment [34, 68] and fluidity [3] in order to successfully host membrane-associating motifs. There are two main types of model membranes: (1) intact vesicle platform (Fig. 3b); and (2) planar lipid bilayer (Fig. 3c). Although they can consist of the same lipid material, the physical properties of the planar bilayer and intact vesicle platforms are quite different.

The planar bilayer presents a flat, uniform surface with its lipid components exhibiting long-range, two-dimensional lateral diffusion [69]. It can self-assemble on certain substrates such as mica and silicon oxide by the surface-induced vesicle fusion mechanism—i.e., adsorption and subsequent rupture of lipid vesicles after reaching a critical vesicle concentration on the substrate [70]. Importantly, the platform is intimately associated with the supporting substrate. Collectively, these features have two effects. First, the planar bilayer is rigidly coupled to the substrate and therefore a thin hydration layer exists between the substrate and lower leaflet [34, 71]. Second, the surface pressure of the bilayer increases when peripheral membrane proteins such as phospholipase A_2_ adsorb to and partially insert themselves into the lipid membrane [72]. This rise in surface pressure can terminate macromolecule adsorption at a lower binding saturation value than that of other model or biological systems [72].

In contrast, the intact vesicle platform is comprised of adsorbed, unruptured lipid vesicles. When spherical lipid vesicles adsorb to substrates such as gold and titanium oxide, they deform but do not rupture [73]. The result is a layer of pancake-like vesicles whose morphology is non-uniform; the intrinsic curvature of this platform surface is important for detecting curvature-sensitive binding of proteins such as those containing BAR domains [74]. The intact vesicle contains hydrodynamically coupled solvent that is encapsulated within its inner core and therefore the layer has viscoelastic properties [75, 76]. As a result, the layer is a soft film that is responsive to the binding of amphipathic macromolecules. Specifically, vesicles can modulate their physical properties in order to maintain a constant surface pressure during the course of a binding interaction [72]. This flexibility enables the determination of binding saturation values that are more representative of biological systems.

In addition to model membranes, sensing platforms can consist of cell-derived membranes that contain a natural assortment of components including lipids, proteins, and glycosaminoglycans [77–79]. While model membranes represent a general platform to study membrane association processes driven by amphiphilic interactions, cell-derived membranes contain receptors which can mediate specific binding of proteins. When studied in conjunction, model and cell-derived membranes can identify the determinants of membrane association for a target protein. Indeed, this approach has significantly aided characterization of the binding dynamics of the NS5A AH in one recent study [80].

**Fig. 4:**
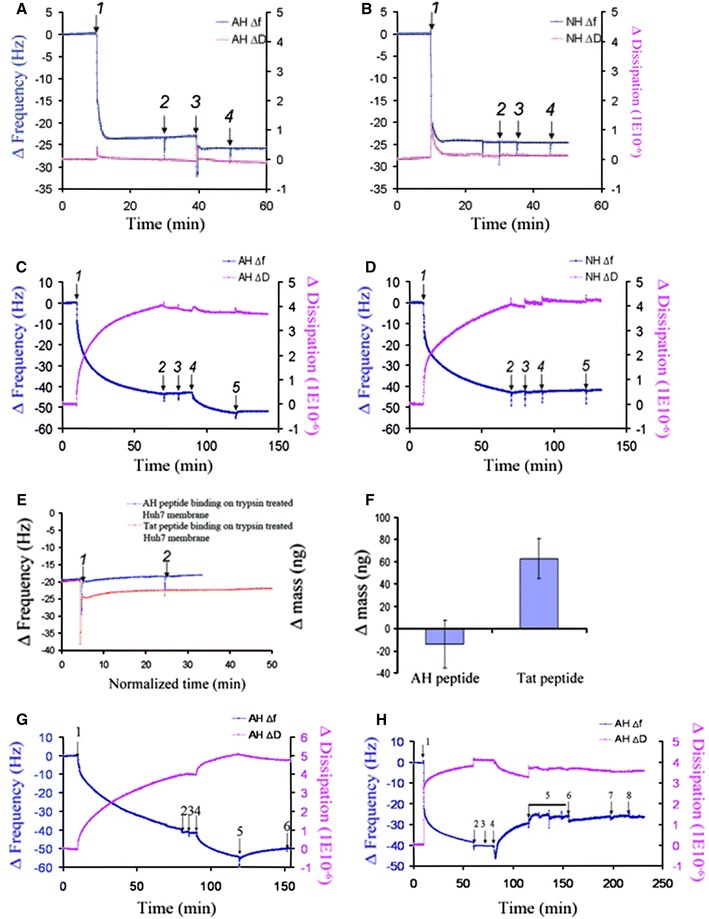
Biosensor strategy to determine NS5A AH binding receptor. QCM-D biosensing detected the interaction of AH peptide with model and Huh7 cell-derived membranes. Changes in resonance frequency of oscillation and energy dissipation were monitored. Binding of AH peptide to model membranes. **a** AH peptide binds to planar lipid bilayers. At 10 min, lipid vesicles were added to a silicon oxide substrate (*arrow 1*). The vesicles fused and ruptured to form a planar bilayer. After a buffer wash at 30 min, AH peptide was injected at 40 min (*arrow 3*). In order to confirm specific binding of the peptide, an additional wash with the same buffer was performed (*arrow 4*). **b** NH peptide does not bind to planar lipid bilayers. Identical experiments to those in **a** were performed, but no binding of the control NH peptide to the planar lipid bilayer was detected. Binding of AH peptide to cell-derived membranes. **c** AH peptide binds to Huh7-derived membranes. At 10 min, Huh7-derived microsomes were injected (*arrow 1*). After buffer washes at 70 min and 80 min (*arrows 2 and 3*, respectively), AH peptide was injected at 90 min (*arrow 4*). In order to confirm specific binding of the peptide, an additional wash with the same buffer was performed (*arrow 5*). **d** NH peptide does not bind to cell-derived membranes. Identical experiments to those in Fig. 6c were performed, but no binding of the control NH peptide to Huh7-derived membranes was detected. Biochemical strategy to confirm proteinaceous receptor preference. **e** Trypsin treatment does not affect the binding of TAT-derived peptide, which binds membranes independently of a protein receptor. AH peptide or TAT-derived peptide was added (*arrow 1*) to trypsin-treated Huh7 membranes deposited on silicon oxide, as in *panel a*, followed by a buffer wash 20 min later (*arrow 2*). **f** Mass changes associated with binding of the AH and TAT-derived peptides to the trypsin-treated Huh7-derived membranes of *panel c*, as calculated using the Sauerbrey equation. QCM-D analysis of monoclonal antibody FG6 binding to its ER membrane receptor PTP1B and sensitivity of binding to prior trypsin treatment of the membranes. **g** Binding of monoclonal antibody FG6 to its ER membrane receptor PTP1B contained in Huh7-derived membranes. At 10 min, Huh7-derived microsomes were injected (*arrow 1*). After buffer washes at 70 min and 80 min (*arrows 2 and 3*, respectively), monoclonal antibody FG6 was injected at 90 min (*arrow 4*). To ensure that the antibody was indeed bound to its membrane receptor, the membranes were washed again with PBS buffer (*arrows 5 and 6*). **h** PTP1B does not bind to trypsin-treated Huh7-derived membranes. At 10 min, Huh7-derived membranes were injected (*arrow 1*), followed by washing twice with PBS buffer (*arrows 2 and 3*, respectively). Then, trypsin was applied to cleave proteinaceous components (*arrow 4*), followed by thorough washes with buffer. After the washing steps were repeated four times (*arrow 5*), the anti-human PTP1B (FG6) antibody was injected (*arrow 6*) to examine its ability to bind to the trypsin-treated Huh7-derived membrane, followed by additional washing steps (*arrows 7 and 8*, respectively). Figure is adapted and modified from Ref. [80]

To monitor membrane-protein interactions in this work, the quartz crystal microbalance with dissipation monitoring (QCM-D) technique was selected. QCM-D enables real-time monitoring of binding kinetics and changes in thin film properties, and is therefore becoming increasingly popular for probing biological interfaces [81–83]. The model membrane platform was first assembled on the sensor chip before monitoring membrane association of the AH peptide. For the model membrane studies, a planar bilayer was self-assembled on silicon oxide [70] (Fig. 4a, b). As expected, the AH peptide demonstrated binding to the planar lipid bilayer (Fig. 4a), whereas the control NH peptide did not bind (Fig. 4b). These experiments were in good agreement with past biological results [63, 67], thus establishing a correlation between standard molecular virology techniques and the QCM-D technology. While the binding of peptides and proteins to model membranes has been demonstrated before [84–86], the study followed up on these initial experiments by developing a novel biomembrane-on-a-chip platform.

Cell-derived membranes from Huh7 cells—a human liver tumor cell line commonly used for HCV replication studies—were assembled on the sensor chip as platforms to probe binding of AH peptide. In this case, significantly more AH peptide associated to the cell-derived membrane as compared to the planar bilayer (Fig. 4c). However, NH peptide once again did not bind to the cell-derived platform (Fig. 4d). These comparison studies with the model and cell-derived membranes suggested that there is a specific binding receptor of the NS5A AH within Huh7 cell-derived membranes.

To confirm this binding specificity, the authors combined biochemical methods with the QCM-D platform. Specifically, pretreatment with trypsin removed proteinaceous components from the Huh7 cell membranes before experiment. When the interactions of AH peptide and transactivator of transcription (TAT)-derived peptide to these trypsin-treated membranes were monitored, only the TAT-derived peptide demonstrated binding (Fig. 4e). The binding mass values were calculated from the QCM-D results and confirmed that only the TAT-derived peptide showed a positive binding mass on the treated membrane platform (Fig. 4f). This discrepancy was expected because the TAT-derived peptide is believed to bind to membranes via extracellular glycosaminoglycans [87], and not protein receptors. By contrast, the sensitivity of the AH peptide binding to trypsin treatment further supports that it specifically interacts with a protein receptor.

In addition to these experiments, one more set of controls was performed to verify the removal of proteinaceous components by trypsin treatment. Protein Tyrosine Phosphatase 1B (PTP1B) is a membrane protein found in Huh7-derived membranes. In the absence of trypsin treatment, antibody binding to PTP1B was observed (Fig. 4g). However, after trypsin treatment, no binding occurred (Fig. 4h). As such, it was verified that trypsin treatment is a valid method to remove proteinaceous components in this assay format. While QCM-D monitoring enabled sensitive detection of membrane-peptide interactions, drawing conclusions from the results of the study required integrated analysis of the biochemical properties of the biomembrane-on-a-chip platform.

Based on this collective set of experiments, the AH peptide demonstrated significantly greater binding to cell-derived versus model membranes. A battery of biochemical control experiments confirmed that this difference is caused by proteinaceous components only found in the cell-derived membranes. By combining the model and cell-derived membrane platforms, the authors were able to support but not confirm the hypothesis that a protein receptor for the NS5A AH resides in the membrane of the host cell endoplasmic reticulum.

## Fundamental Characterization of Model Biological System

To further probe membrane association of the NS5A AH, there are several characterization strategies that can be taken based on the observation that proteinaceous components influence the binding of AH peptide to lipid membranes in one of two ways. First, the AH peptide ligand may directly bind a specific protein receptor of the host cell, as proposed in the initial hypothesis. Second, proteinaceous components may modulate the properties of the lipid membrane itself, thus affecting the binding interaction of the AH peptide to lipid and/or other protein components. Because the AH peptide is amphipathic, physical properties of the lipid membrane are likely to play a significant role in this interaction. While the AH peptide demonstrated minimal binding to a planar bilayer, it is important to determine whether this interaction kinetics holds for all types of model membranes or whether it is the result of the platform design. As previously discussed, there are other useful model membrane platforms to mimic cell membranes, including the intact vesicle platform which is a soft film that is more biologically relevant for studying macromolecular interaction kinetics.

Recent studies have investigated how AH peptide affects the film properties of the intact vesicle platform [40, 42]. With the collection of characterization data, there is an increasing amount of information about the molecular determinants of the target interaction. By understanding these parameters, the overall process of fundamental characterization can identify potential biomedical applications, and guide translational research directed at validating these applications. This work is an excellent example of how engineering strategies can reveal unexpected findings into the mechanisms of biological interactions.

### Vesicle-to-Bilayer Structural Transformation by AH Peptide

**Fig. 5:**
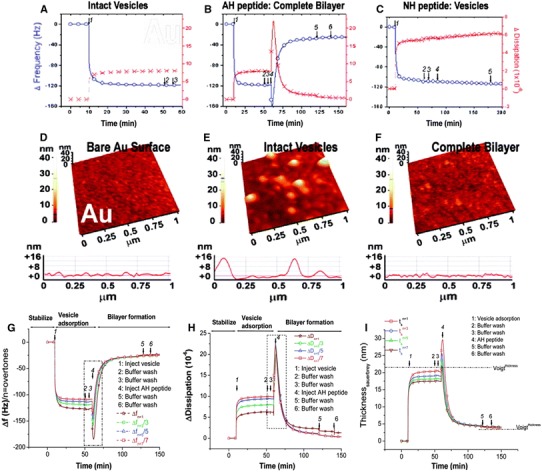
Identification of vesicle rupturing process by AH peptide. AH peptide-vesicle interaction analysis. Changes in resonance frequency of oscillation (*blue curve*) and energy dissipation (*red curve*) were monitored by the QCM-D technique. All data were collected at the third overtone and normalized. **a** Intact vesicle adsorption on an oxidized gold surface. After 10 min of stabilization, lipid vesicles were added (*arrow 1*). After 50 and 55 min, buffer washes were performed in order to test the stability of the film (*arrows 2 and 3*, respectively). **b** AH peptide promotes vesicle-to-bilayer transformation. At 60 min (*arrow 4*), AH peptide was added to the intact vesicle layer on the gold surface. After 120 and 140 min, buffer washes were again performed (*arrows 5 and 6*, respectively). **c** NH peptide has no effect on the intact vesicle platform. Identical experiments to those described in Fig. 8b were performed with the control NH peptide, and demonstrated that the NH peptide does not affect the properties of the intact vesicle layer. Morphological effects of AH peptide-mediated vesicle rupture. AFM imaging characterized the vesicle rupturing process on gold. All images are presented in height mode. **d** Oxidized gold surface. **e** Adsorbed layer of intact lipid vesicles on the gold surface. **f** Planar bilayer self-assembles after treatment with the AH peptide. Modeling QCM-D responses for structural transformation from adsorbed vesicles to a planar bilayer. Identical experiments to those described in Fig. 8b were performed. **g** Resonance frequency responses at multiple overtones were recorded for the vesicle-to-bilayer transformation. **h** Corresponding energy dissipation responses were also recorded. **i** Change in film thickness was determined by application of the Sauerbrey equation to the QCM-D data. For comparison, we also present the thicknesses calculated from the Voigt-Voinova model for the intact vesicle layer (22 nm) and planar bilayer (4.4 nm). Note that *n* = 1 shows fundamental overtones (*open squares*), *n* = 3 shows third overtones (*open circles*), *n* = 5 shows fifth overtones (*open triangles*), and *n* = 7 shows seventh overtones (*opposite triangles*). Figure is adapted and modified from Refs. [40, 42]

To characterize the interaction of AH peptide with the intact vesicle platform, QCM-D monitoring and atomic force microscopy (AFM) were employed as complementary techniques to respectively probe the interaction kinetics and morphological changes [82, 88]. The adsorption of lipid vesicles onto solid supports results in the self-assembly of different lipid structures. As shown in the previous example, lipid vesicles adsorb and rupture on silicon oxide to form a planar bilayer. By contrast, lipid vesicles adsorb but do not rupture on gold [76]. Instead, the intact vesicles form a stable layer. QCM-D measurements demonstrated that this self-assembly process follows exponential adsorption kinetics, and results in the formation of a stable sensing platform (Fig. 5a). Strikingly, upon addition of AH peptide to the vesicle platform, the intact vesicle layer transformed into a planar bilayer (Fig. 5b). Control experiments with the NH peptide demonstrated that the amphipathic, α-helical character of the AH peptide is a key determinant of its vesicle-rupturing ability (Fig. 5c) The structural transformation process was also followed directly by AFM imaging in order to measure the height profile of the bare gold surface (Fig. 5d), intact vesicle layer (Fig. 5e), and planar bilayer (Fig. 5f). This analysis confirmed the formation of a planar bilayer on gold.

While vesicle rupturing is typical of membrane lysis processes catalyzed by peptides [89, 90], this result is particularly exciting because it is the first example of a biological interaction that promotes the structural transformation of a viscoelastic layer to form a non-viscoelastic film. By contrast, an earlier example identified by Hook et al. demonstrated the structural transformation of a mussel adhesive protein adlayer, but this process was induced by chemical reaction with NaIO_4_ to cross-link a protein film [91]. The case of the AH peptide has direct biological relevance because membrane association of the NS5A AH is necessary for HCV genome replication. Critically, this strategy also solves a major design challenge for fabricating planar bilayers on gold, which has a number of attractive electrical and optical properties.

In another study, the AH peptide-mediated process of vesicle rupture and planar bilayer formation was fit with a the Voigt-Voinova based model to analyze how the peptide interaction alters film properties including thickness, effective shear modulus, and shear viscosity [92]. To fit the model, QCM-D resonance frequency and energy dissipation measurements of the AH peptide-mediated structural transformation were recorded at multiple overtones (Fig. 5g, h). Film thicknesses calculated by the Voigt-Voinova model confirmed that the Sauerbrey relationship—which describes the simple linear relationship between change in resonance frequency and mass adsorption in limited cases—is only valid for analyzing rigid films, and not soft layers (Fig. 5i). The results of this modeling confirm the importance of collecting a wide range of data. In this case, additional information enabled the change in mechanical properties of the thin film to be correlated with the AH peptide-membrane interaction. This combined approach may also be helpful for studying the mechanism of AH peptide’s vesicle-rupturing activity.

Based on the results of these studies, we observe several key findings. First, the maximum binding signal generated by adsorption of AH peptide to the intact vesicle platform is significantly greater than the signal on the planar bilayer platform. In part, this discrepancy may be due to differences in the structural features of a planar bilayer versus layer of intact vesicles. Indeed, in a fixed geometry, intact vesicles would have more available surface area for peptide binding than the planar bilayer. However, when the binding signals are normalized to account for the varying amount of lipid material in the different platforms, the binding signal on the intact vesicle platform is still 2.5 times greater. Depending on the target interaction, this platform design issue may be an important consideration for biosensing.

Second, the interaction kinetic are completely distinct in each case. On the planar bilayer platform, simple exponential adsorption kinetics is observed with minimal peptide binding. This behavior suggests that the interaction is nonspecifically mediated by the amphipathic nature of the peptide, and that membrane-associated AH peptide exists in the monomeric state. By contrast, interaction of AH peptide with intact vesicles promotes a structural transformation from intact vesicle layer to planar bilayer. The interaction kinetics are complex and show a two-step process. While the Voigt-Voinova model helped to determine the effects of the transformation process on film properties, additional evidence is necessary to understand the mechanism of this process. In this regard, recent work by Hatzakis et al. [93] on the intrinsic property of bilayers to recruit larger densities of amphipathic molecules when highly curved deserves attention. In addition, as the intact vesicle layer is the more biologically relevant model membrane system, the interaction kinetics observed in this system may be related to the role of NS5A protein in the HCV life cycle.

Third, the concentration-dependence of the AH peptide interaction varies between the platforms. At a concentration of 0.05 mg/mL, the AH peptide transforms a layer of intact vesicles into a planar bilayer. However, at the same concentration, the AH peptide does not bind to the planar bilayer. Higher concentrations of AH peptide are necessary to detect any binding signal on the planar bilayer platform. Taken together with the QCM-D responses, these data support that the transformation of an intact vesicle layer on titanium oxide or gold can result in the formation of a complete, planar bilayer with comparable sensing properties to those of a planar bilayer on the prototypical silicon oxide substrate. From an applications standpoint, the fabrication of a planar bilayer on these formerly intractable substrates is advantageous for the design of optical- and electrochemical-based sensors.

### Multiple Technique Characterization of AH Peptide-Mediated Vesicle Rupture

While the assembly of a planar bilayer on gold and titanium oxide represents an important step towards the application of biological tools to solve problems in materials science, a more complete description of the fundamental mechanism behind the transformation process requires detailed study from multiple angles in order to evaluate its biomedical significance. Indeed, complex biological phenomena are difficult to interpret based on a single physical principle. In the case of the interaction between AH peptide and lipid vesicles, the QCM-D energy dissipation signal associated with the vesicle-to-bilayer transformation indicated an intermediate regime of high viscoelasticity. Such a change in film viscoelasticity might occur due to an increase in bound solvent resulting from vesicle swelling, for example. However, the QCM-D technique only senses acoustic mass, which is the combined mass of the adsorbed material (e.g., lipid and/or amino acid) as well as bound solvent. On the basis of this measurement alone, it is not possible to identify how the solvent mass fraction changes during the transformation. To address this challenge, it is therefore important to characterize biointerfacial processes by using several surface-sensitive techniques that measure different physical principles.

To investigate the mechanism by which AH peptide modulates the film properties of the intact vesicle layer, simultaneous QCM-D and reflectometry measurements to follow the AH peptide-mediated vesicle-to-bilayer transformation on gold have been reported [43]. While the acoustic wave-based QCM-D technique characterizes the structural properties of an adlayer film, the optical-based reflectometry technique provides a measure of the bound molecular mass that is related to the effective thickness and the effective refractive index of the adlayer [94]. When these two techniques are combined, several parameters can be simultaneously measured as a function of time, including: (1) adsorbed molecular mass (optical mass); (2) adsorbed molecular mass and solvent mass (acoustic mass); (3) effective film thickness; and (4) effective refractive index over the course of time [94]. As a result, the solvent mass can be calculated by separating the acoustic and optical masses. Because vesicle swelling and expansion was initially hypothesized to be the cause of the AH peptide-mediated vesicle rupturing process, the ability to follow the change in solvent mass is particularly important to test this hypothesis and further understand the biological mechanism.

**Fig. 6:**
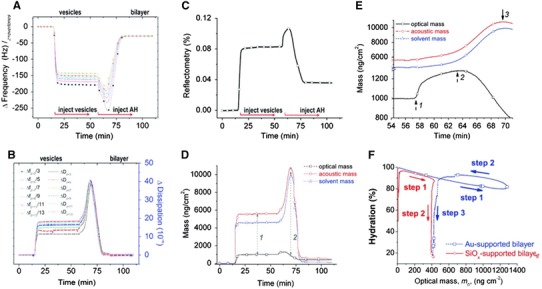
Mechanism of AH peptide interaction with lipid vesicles. Combined QCM-D and reflectometry analysis for AH peptide-mediated structural transformation from intact vesicle layer to planar bilayer on a gold surface. **a** QCM-D resonance frequency data of structural transformation process. Multiple overtones, z, up to the 13th, were recorded. **b** QCM-D energy dissipation data of structural transformation process. The data demonstrate the changes in viscoelasticity of the film during the vesicle-to-bilayer transformation. **c** Simultaneously, the reflectometric response was also obtained. **d** Acoustic mass (*red long-dotted line*) and optical mass signatures (*black line*) of the structural transformation can elucidate the mass contributed by bound solvent (*blue dot*). Parameters used for calculating the optical mass and solvent mass were *ρ*_solvent_ = 1.0 g m^−3^, *r* = 0.286 cm^3^ g, *ρ* = 1.02 g cm^−3^, *v* = 0.98 cm^3^ g^−1^, and *n*_0_ = 1.334. **e** Magnified view of the graph in part d. Note that there is a break on the *y*-axis from 1,290 to 4,500 ng/cm^−2^, the *lower* and *upper* scales differ by a factor of 15, and the data are only plotted from 54 to 70 min. **f** Hydration signatures of vesicle rupture on gold (Au) and silicon oxide (SiO_x_). The hydration signature helps to describe the mechanism of structural transformation processes. Figure is adapted and modified from Ref. [43]

Based on this combined measurement approach, the vesicle-to-bilayer structural transformation process was followed by QCM-D resonance frequency (Fig. 6a) and energy dissipation signals (Fig. 6b). The reflectometry signal was simultaneously recorded on the same surface (Fig. 6c). By using a number of formalisms, the QCM-D and reflectometry data can be converted to acoustic and optical mass terms that describe the physical properties of the adsorbed film. Changes in the acoustic, optical, and solvent masses of the film were followed as a function of time (Fig. 6d). This analysis identified four regimes to describe the transformation process: (1) soft, intact vesicle layer; (2) initial interaction of AH peptide with vesicle layer; (3) vesicle rupturing; and (4) rigid, planar bilayer.

A magnified view of Fig. 6d is presented to highlight several key observations about the regimes of initial peptide interaction and vesicle rupturing (Fig. 6e). First, selection of the surface-sensitive technique is critical for detection of the target interaction. Only minor structural changes were detected by the QCM-D technique upon initial binding of AH peptide. By contrast, reflectometry was able to clearly identify and quantify this binding step. Second, saturation of AH peptide binding occurred before the swelling of vesicles reached saturation. This lag time of approximately 6 min suggests that membrane association of the AH peptide is insufficient to directly cause swelling and vesicle destabilization. Rather, some type of peptide rearrangement (e.g., pore formation) may occur to induce solvent uptake. Third, the optical mass measurement provides direct evidence that AH peptides bind to lipid vesicles.

Another benefit of the combined measurement approach is the ability to calculate a hydration signature to describe the role of solvent mass throughout the course of the interaction. To demonstrate how this parameter is useful for comparing macromolecular interactions, the self-assembly processes for planar bilayer formation on silicon oxide and gold were compared (Fig. 6f). An especially useful feature of hydration signature is the direct identification of individual kinetic steps to describe the overall mechanism of biological interactions [43, 95].

Taken together, the combination of QCM-D and reflectometry techniques enabled a more complete description of the AH peptide-mediated vesicle rupturing process. Large increases in adsorbed mass and energy dissipation are caused by swelling of the vesicles upon interaction with AH peptide. Based on the kinetic differences of the optical and acoustic mass responses, it appears that the binding of AH peptide is necessary but not sufficient to promote the structural transformation. Instead, the AH peptides presumably rearrange on the membrane interface to promote vesicle swelling and destabilization, leading to reassembly and planar bilayer formation. With these mechanistic details revealed, there is now a framework in place to evaluate the biomedical significance of this interaction and to engineer optimized peptides with more potent rupturing activity.

## From Engineering to Biomedical Application

Based on the steps of platform design and fundamental characterization, the lipid vesicle-rupturing ability of the NS5A AH peptide was identified. Beyond the biological significance of this activity which remains to be determined, there are several important applications which directly emerge from this activity. First, there is now a strategy to form planar bilayers on previously intractable substrates that are important materials for biosensing. Second, AH peptide-mediated vesicle rupturing is a targetable activity of the NS5A protein that may serve as a surrogate for targeting its membrane association step and/or other biologically significant function. Third, the AH peptide demonstrates potential for rupturing lipid-enveloped virus particles that share similar membrane properties to lipid vesicles. In terms of direct impact, the virus particle-rupturing potential of the AH peptide is greatest because this inhibition strategy may serve as a broad-spectrum activity against all lipid-enveloped viruses or some subset thereof. Further, this rupturing activity would represent a completely novel mechanism of action that targets a conserved structural feature of the virus particle that is not encoded by the viral genome.

### Vesicle Rupture as a Predictor of Virus Particle Lysis

To determine if the AH peptide possesses suitable properties for rupturing the lipid envelopes of virus particles, it is necessary to develop an appropriate model system to mimic the envelope structure. In this sense, intact lipid vesicles are an excellent choice because they replicate the membranous environment of lipid envelopes [50, 96–100]. Unlike the planar bilayer, intact vesicles also recapitulate membrane curvature. While the mechanism of the AH peptide’s vesicle rupturing activity was already determined by the combined QCM-D and reflectometry measurements, it was not determined how this in situ vesicle rupturing activity may correlate with in vitro virus particle rupturing.

Initial studies on the AH peptide-mediated rupturing process focused on vesicles with 50–60 nm diameters. This size range was selected because zwitterionic lipid vesicles of this diameter easily rupture on silicon oxide [101]. For fundamental characterization of surface-specific adsorption kinetics, the vesicle size was therefore fixed and the variable parameter was the substrate material. This characterization work eventually led to the development of the planar bilayer and intact vesicle platforms which were used for probing AH peptide-mediated vesicle rupturing. In general, the different film properties of these two platforms are sufficient for studying how membrane properties affect these interactions. However, to further evaluate the AH peptide, the intact vesicle platform should be modified to better mimic the properties of lipid-enveloped virus particles. One of the key differences between the particles of different viruses is their size distribution, which is therefore a useful parameter to study. Indeed, one study recently used the intact vesicle platform with the QCM-D assay to determine the vesicle size range within which the AH peptide demonstrates rupturing activity in order to evaluate which viruses the AH peptide may target.

**Fig. 7:**
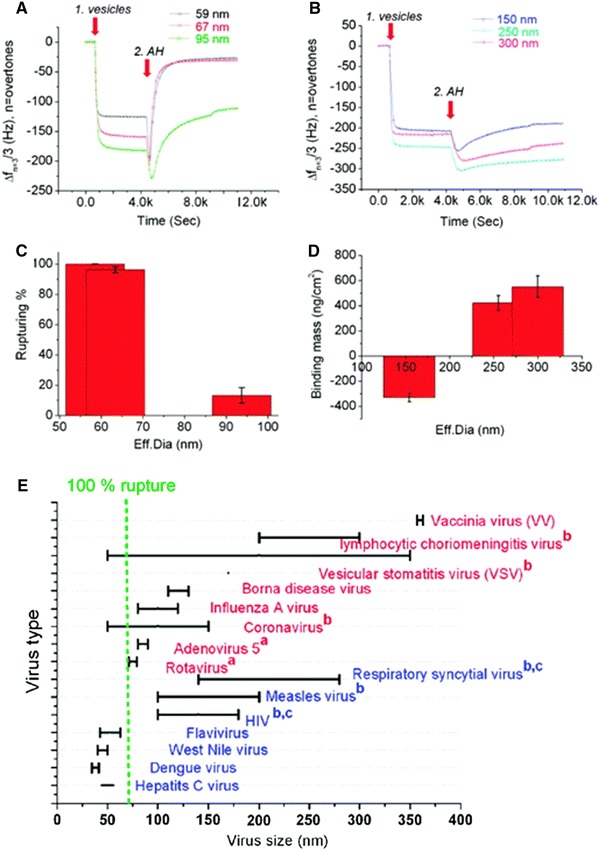
Biophysical characterization of AH peptide-mediated vesicle rupturing. The antiviral mode of action of the AH peptide was studied with the intact vesicle platform on the QCM-D sensor. **a** Resonance frequency response for different vesicle size distributions up to 100 nm upon AH peptide addition. Vesicles were added at 1.0 ks (*arrow 1*), and AH peptide was added at 4.2 ks (*arrow 2*). **b** Resonance frequency response for different vesicle size distributions greater than 100 nm upon AH peptide addition. Again, vesicles were added at 1.0 ks (*arrow 1*), and AH peptide was added at 4.2 ks (*arrow 2*). Note that the average vesicle effective diameters were measured by dynamic light scattering. **c** Calculation of rupturing efficiency from the resonance frequency response as a function of lipid vesicle size. One hundred percent rupturing is defined as the complete transformation of the intact vesicle layer into a planar lipid bilayer with the characteristic final frequency change of −26 Hz. **d** Interaction of AH peptide with large vesicles beyond the rupturing range. The absolute change in bound mass is determined from the data presented in Fig. 7b. **e** Broad-spectrum virocidal potential of AH peptide. The graph represents data replotted from Ref. [45], wherein the size distribution of virus particles is indicated by the *black bars*. Susceptibility to AH peptide-mediated inhibition of infectivity is indicated for a variety of viruses, as determined in Ref. [45]. Viruses reported to be susceptible and refractory to AH peptide-mediated treatment are indicated in *blue* and *red*, respectively. The *vertical green dotted line* indicates the size cutoff below which 100% lipid vesicle rupture occurs. Figure is adapted and modified from Ref. [41]

The QCM-D measurements identified three regimes to describe the process of AH peptide-mediated vesicle rupture: (1) complete planar bilayer formation for vesicles with an average diameter less than 70 nm; (2) incomplete rupture of vesicles with an average diameter greater than 90 nm; and (3) minimal to no rupture of vesicles with diameters significantly larger than 90 nm (Fig. 7a, b). Based on these results, an evaluation criterion, the degree of vesicle rupturing, was established to assess the rupturing activity of AH peptide against lipid vesicles of varying sizes (Fig. 7c). The rupture percentage provides a general, quantitative framework to measure the performance of other candidate antivirals which target lipid envelopes as well as to identity compounds which abrogate AH peptide’s activity. For larger vesicles with an average diameter of at least 150 nm, the binding mass of the AH peptide to the vesicle layer was calculated by the Sauerbrey relationship (Fig. 7d). In this size range, the predominant interaction is AH peptide binding rather than vesicle rupture.

Taken together, these data demonstrate that the AH peptide possesses a selective targeting of small virus particles. Based on the results, a graph was constructed and identified that several Flaviviridae members including HCV are likely to be targeted by the AH peptide (Fig. 7e). Atomic force microscopy (AFM) measurements confirmed that AH peptide treatment can physically disrupt the lipid envelopes of viruses, including HCV, only within the target size range. While fusion peptides derived from viral envelope proteins have previously been shown to display antiviral activity by either destabilizing lipid envelopes or interfering with fusogenic domains of envelope proteins [102, 103], the AH peptide is neither involved in membrane fusion nor known to interact with other protein domains within the HCV NS5A protein. Furthermore, the NS5A protein is a nonstructural protein that is involved in intracellular HCV genome replication, and is not part of the HCV virion. Several antimicrobial peptides have also been described that physically disrupt viral envelopes [104].

By directly monitoring the physical effect of AH peptide treatment on enveloped viruses as well as confirming these findings with viral replication monitoring, the study demonstrated that the intact vesicle platform represents a reliable model system for predicting the susceptibility of individual viruses to AH peptide treatment. Predictions are most valid for viruses that more closely approximate key properties of synthetic lipid vesicles; including: (1) have a lipid envelope; and (2) minimal heterogeneity in particle size. Furthermore, it is not likely that the size corresponding to 100% vesicle rupture is the maximum size at which AH peptide can rupture virus particles. A more probable case is that the rupturing ability of the AH peptide against virus particles occurs on a continuum, as observed in Fig. 7c. Therefore, while the AH peptide may have a greater observed effect on smaller virus particles, it more likely still interacts with larger particles and may affect their infectivity even in the absence of direct rupturing. In addition to this method focused on determining the biophysical mechanism of the AH peptide-mediated vesicle rupturing process in order to predict therapeutic effect, other groups have followed up by confirming the in vitro activity of the AH peptide with molecular virology approaches.

### Broad-Spectrum Virocidal Activity of AH Peptide

**Fig. 8:**
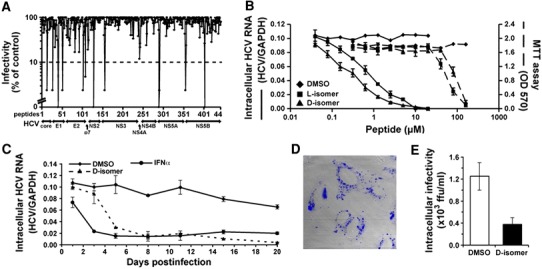
Inhibition of HCV infection by AH peptide analogue. **a** A library consisting of overlapping 18-mer peptides from the entire HCV polyprotein was screened for the ability to inhibit HCV infection in a focus reduction assay using Huh-7.5.1 cells. The peptide from the N-terminal region of NS5A displayed the strongest inhibition. **b** Determination of peptide concentration required to inhibit HCV infection by 50% (IC_50_). Peptide stock solutions were serially diluted in DMSO and tested for inhibitory activity. Peptide cytotoxic activity was measured MTT cytotoxicity assay. The peptide concentration that reduced the cell growth by 50% was designated as the LC_50_. **c** AH peptide analogue prevents initiation of HCV infection and suppresses established infection. Fifteen days after infection of Huh-7 cells, the *l*- and *d*-isomers of the AH peptide analogue were added. At the indicated time points, total cellular HCV RNA content was measured. For comparison, the infected cells were treated with 100 U/mL of recombinant human IFNα. **d** AH peptide analogue inhibits intracellular HCV particle infectivity. To determine whether the peptide enters cells, a fluorescently labeled version was incubated with Huh-7 cells, and analyzed by confocal fluorescence microscopy. **e** Huh-7 cells previously infected with HCV were treated with *d*-isomers of AH peptide analogue or DMSO. After 6 h, intracellular HCV infectivity was determined. Figure is adapted and modified from Ref. [45]

While screening HCV-derived peptides capable of inhibiting HCV viral entry, Cheng et al. [45] serendipitously discovered the AH peptide’s virocidal activity. The peptide from the NS5A N-terminal AH anchor region—termed C5A—had the strongest effect on inhibiting HCV infection (Fig. 8a). For clarification, note that the C5A peptide is an 18 amino acid fragment of the full 27 amino acid long N-terminal AH peptide which our team originally described. More detailed analysis revealed that C5A inhibited HCV infection at submicromolar, noncytotoxic concentrations (Fig. 8b). This finding is very important because certain antimicrobial peptides such as dermaseptin and tachyplesin display inhibitory activity against some enveloped viruses, albeit they are not broad-spectrum antivirals and their development is limited as the result of cytotoxicity issues [104]. Compared to daily treatment with recombinant human interferon-alpha (IFNα)—the standard HCV treatment option [105]—C5A inhibited intracellular HCV RNA accumulation almost as effectively by day 5 (Fig. 8c). Importantly, C5A outperformed IFNα on days 15 and 20 of treatment, suggesting that the peptide can halt persistent HCV infection in growth-arrested cells.

Another important feature identified by the authors was that C5A peptide can penetrate cell membranes (Fig. 8d) and has intracellular virocidal activity (Fig. 8e). Notably, there was no change in the amount of intracellular HCV RNA, confirming our finding that the AH peptide directly ruptures HCV particles. One point to note the report by Cheng et al. is that the peptide’s extracellular virocidal activity is more efficient than its intracellular activity. Bioavailability of peptide therapeutics is indeed a general challenge for effective drug concentrations to reach intracellular targets and may ultimately direct the peptide to extracellular applications such as prophylaxis. Nonetheless, in vivo studies in mice supported that the peptide has a promising toxicity profile and is non-immunogenic.

In addition to targeting HCV, additional infectivity assays demonstrated that AH peptide has broad-spectrum activity against HIV and additional members of the Flaviviridae and paramyxoviruses—all of which have lipid-enveloped virus particles [96–98]. Most importantly, as shown in Table 2, these molecular virology results support the mechanism of action which was identified based on engineering strategies. In particular, the AH peptide directly targets lipid-enveloped virus particles within a certain size range. Indeed, all viruses that are targeted by the AH peptide have average particle size diameters near or below the 100% vesicle rupturing value measured by the QCM-D assay. By contrast, AH peptide had no effect against virus particles without lipid envelopes or whose particle size diameters were much larger than the 100% rupturing value.

**Table 2:** Virus susceptibility to AH peptide analogue treatment

Virus	Enveloped	Genome	IC_50_, μM	Target range
HCV genotypes				
HCV(JFH-I) genotype 2a	Yes	RNA+	0.6	Yes
HCV (H77 envelope) genotype 1a	Yes	RNA+	3.9	Yes
HCV (Con1 envelope) genotype 1b	Yes	RNA+	1.6	Yes
HCV (J6CF envelope) genotype 2a	Yes	RNA+	1.1	Yes
Other viruses				
Dengue virus	Yes	RNA+	2.0	Yes
West Nile virus	Yes	RNA+	4.5	Yes
Measles virus	Yes	RNA−	2.7	No
Respiratory syncytial virus	Yes	RNA−	4.5	No
Human immunodeficiency virus	Yes	RNA+	1.3	No
Adenovirus	No	DNA	>18	No
Borna disease virus	Yes	RNA−	>18	No
Coronavirus 229E	Yes	RNA+	>18	No
Coxsackie virus	No	RNA+	>18	No
Hepatitis B virus	Yes	DNA	>18	No
Influenza virus	Yes	RNA−	>18	No
Lymphocytic choriomeningitis virus	Yes	RNA−	>18	No
Rhinovirus	No	RNA+	>18	No
Rotavirus WISC2	No	dsRNA	>18	No
Vaccinia virus	Yes	DNA	>18	No
Vesicular stomatitis virus	Yes	RNA−	>18	No

To determine whether the antiviral activity of the AH peptide analogue is specific to HCV, Cheng et al. investigated whether the peptide also inhibited the infectivity of other viruses. Treatment with peptide had no significant effect on the infectivity of adenovirus, Borna disease virus, coronavirus, coxsackie virus, influenza A virus, lymphocytic choriomeningitis virus, rhinovirus, rotavirus, vaccinia virus, or vesicular stomatitis virus, or on the antigenicity and DNA content of hepatitis B virus. In contrast, peptide treatment strongly inhibited the infectivity of chimeric viruses containing the envelope proteins of HCV1a and 1b genotypes, and of other human Flaviviridae members, including West Nile virus and dengue 2 virus. Further, infectivity of the paramyxoviruses, measles and respiratory syncytial virus, and HIV-1 were inhibited by the AH peptide analogue. For each virus type, we report (i) presence of lipid envelope, (ii) type of genetic material, (iii) IC_50_ value of AH peptide analogue, and (iv) whether or not the particle size distribution falls within the range of 100% rupture efficiency by AH peptide. Table is adapted and modified from Ref. [45]

Notably, some viruses including Influenza virus and Borna disease virus possess lipid envelopes and have particle size diameters near the 100% rupturing value, yet are not susceptible to treatment with AH peptide [99, 100]. More work is needed to fully elucidate the molecular determinants of virus susceptibility. Because many of the susceptible viruses such as HCV, West Nile virus, and dengue virus bud into the endoplasmic reticulum (ER) membrane, Cheng et al. attempted to explain their results by proposing, but not verifying, that the AH peptide targets membranes of certain lipid compositions. By contrast, our model provides conclusive evidence that the critical parameter for virus inhibition is the size of lipid-enveloped particles. Nonetheless, the composition of lipid envelopes may be a secondary factor and could be easily tested with the model membrane platform.

In summary, recent virological studies have confirmed that in situ characterization of AH peptide’s vesicle rupturing activity is a reliable predictor of in vitro virocidal activity against lipid-enveloped virus particles [44, 45]. In doing so, the key parameters of the AH peptide’s virocidal activity were found to be that: (1) amphipathic sequence is necessary; (2) lone cysteine residue is also necessary and may be involved in disulfide bond formation; and (3) variation in amino acid composition of the C5A peptides from the different HCV genotypes correlates with varying levels of virocidal activity. In terms of optimization, adjustment of the AH peptide sequence composition will therefore be useful to modulate antiviral potency and therapeutic index [46, 106, 107]. Indeed, Li et al. [106] recently described the rational design of a second-generation peptide that preserves potent anti-HCV and anti-HIV activities while minimizing hemolytic activity. Collectively, the results achieved thus far demonstrate the potential of engineering strategies for the characterization and targeting of membrane-associated steps in viral life cycles.

## Outlook on Engineering Strategies for Antiviral Drug Development

The application of model membrane platforms to target the HCV NS5A protein has led to discovery of the broad-spectrum antiviral activity exhibited by AH peptide. As part of this process, fundamental characterization of the AH peptide-membrane interaction yielded a set of estimations for AH peptide’s virocidal activity, including the size range of lipid-enveloped virus particles which may be targeted. This activity was then validated by molecular virology studies and the AH peptide is now in the drug pipeline. In this outlook, we introduce areas where we envision engineering strategies may further aid antiviral drug development.

By assembling an in situ assay to predict virus particle rupture [40–43], the work reviewed here has established a cost-effective tool to rapidly screen and functionally characterize antiviral drug candidates in minutes rather than days. Based on this preliminary format, the system can be improved by increasing sensitivity to reduce the amount of peptide material required and/or adapting the signal response to a more readily high-throughput measurement technique.

As one alternative assay possibility, multiple studies have reported that the AH peptide can rupture the membranes of lipid vesicles, as observed by the release of encapsulated fluorescent dye [45, 108]. However, this assay would first need to be standardized before functional characterization and screening. Thus far, the reports describing this assay have used different lipid compositions, fluorescent dye reagents, and concentrations thereof. Further, the vesicle preparation methods have varied and there has been no physical characterization of vesicle properties. All of these parameters would affect the measurement response, and need to be controlled. These considerations raise awareness of the challenges for studying the biointerfacial sciences, and further demonstrate how engineering strategies such as those described here can streamline drug development and increase reproducibility and accuracy.

A significant benefit of the established QCM-D vesicle rupturing assay is its ability to directly characterize the physical properties of the intact vesicle platform. By this method, any major differences in the film property can be identified before experiment and minor differences can be normalized. Real-time monitoring of the interaction kinetics yields a distinct, complex signature based on a self-assembly process. As such, it is difficult to generate a false positive due to the complex signature, and yet it is also still practical to screen for this signature in a high-throughput format because it leads to a clear result, the self-assembly of a planar bilayer on gold with well-characterized mass and viscoelastic properties. At present, it is not known if there would be any correlation between the results of the QCM-D assay and a fluorescence-based membrane rupturing assay. Nonetheless, continued innovation in these types of functional assays will help to speed up drug discovery and development.

One area where these engineering assays would be useful is the characterization of other classes of antiviral drug candidates. Beyond lipid envelope-rupturing peptides, a small molecule compound has been reported to physically disrupt lipid membranes [109]. Interestingly, this compound displays a general property of membrane disruption but only permanently damages the membranes of viruses and not host cells. Unlike the host cell which maintains a biogenic capacity to repair its lipid membranes [110], viruses do not have such reparative capacity [111–113]. As a result, this compound has a low toxicity profile and the chemical structure is amenable to optimization for improved pharmacokinetics. Along with the new class of phospholipid-mimicking RAFIs (Rigid Amphipathic Fusion Inhibitors) [114] and Arbidol—a broad-spectrum small molecule antiviral available in Russia and China [115–118]—the biophysical mechanism of these fusion inhibitors remains to be determined.

In terms of looking forward, we also believe that the NS5A-encoded AH represents a unique target. In addition to the zinc-binding domain of NS5A [66], the N-terminal AH is the sole, validated functional target. With the promising results of a recent Phase I clinical trial for the NS5A-targeting BMS-790052 compound against HCV infection in patients who followed a once-daily, dosing regimen [119], there is huge potential for developing additional inhibition strategies against the NS5A protein. Indeed, the amphipathic character of the AH is conserved across all HCV isolates to date [63, 64, 67], making the AH far less likely to evolve resistance mutations [120, 121]. The QCM-D vesicle rupturing assay could be alternatively used to screen chemical libraries for small molecules that inhibit vesicle rupturing by the NS5A AH peptide. On the same token, the broad-spectrum antiviral activity of the NS5A AH peptide itself is very attractive for many therapeutic uses, including as a broad-spectrum antiviral agent or even as an immune adjuvant [107].

In summary, engineering strategies offer a new approach to probe biological systems by designing physiologically relevant model platforms through bottom-up design. While the focus here has been on antiviral drug development, model membranes offer an excellent platform to study a wide range of membrane-associated biological processes. Since many of these activities are critical determinants of human health, it will be exciting to witness the application of these strategies to solve biomedical problems that are currently intractable.
